# The role of syndromic knowledge in Ethiopian veterinarians’ treatment of cattle

**DOI:** 10.3389/fvets.2024.1364963

**Published:** 2024-08-30

**Authors:** Ndungu S. Nyokabi, James L. N. Wood, Gizachew Gemechu, Stefan Berg, Adane Mihret, Johanna F. Lindahl, Henrietta L. Moore

**Affiliations:** ^1^Institute for Global Prosperity, University College London, London, United Kingdom; ^2^University of Edinburgh Business School, Edinburgh, United Kingdom; ^3^Department of Veterinary Medicine, University of Cambridge, Cambridge, United Kingdom; ^4^Armauer Hansen Research Institute, Addis Ababa, Ethiopia; ^5^Bernhard Nocht Institute for Tropical Medicine, Hamburg, Germany; ^6^International Livestock Research Institute (ILRI), Nairobi, Kenya; ^7^Department of Medical Biochemistry and Microbiology, Uppsala University, Uppsala, Sweden; ^8^Department of Clinical Sciences, Swedish University of Agricultural Sciences, Uppsala, Sweden

**Keywords:** animal health services, cattle diseases, disease surveillance, zoonoses, biosecurity practices, one health, herd health management, dairy cattle

## Abstract

Veterinarians play a significant role in the treatment and prevention of livestock diseases at the farm level, safeguarding public health and ensuring food safety. In sub-Saharan Africa, access to quality veterinary services is a major challenge for livestock farmers due to the low number of publicly employed veterinarians, underfunding and privatisation of veterinary services. Low investment in veterinary services and infrastructure, including a lack of laboratories for diagnosis, has made veterinarians rely on their experience and knowledge of cattle disease symptoms developed over years of practice to diagnose and treat cattle diseases. A cross-sectional survey using a role-play approach was used to collect data on knowledge regarding cattle diseases among veterinarians in veterinary clinics and private practices in Addis Ababa, Oromia and Adama regions in Ethiopia. Veterinarians were given a number of disease scenarios based on “fictive disease symptoms” that are commonly manifested in a sick cow and asked to identify the disease what personal biosecurity they would use, diagnostic tests they would perform, treatments they would prescribe, treatment costs, and additional services and inputs they would recommend to the farmer. The results show that veterinarians could identify endemic cattle diseases through symptoms. The majority of veterinarians did not find it important to report notifiable diseases, a behaviour which could hamper disease surveillance and outbreak response. The advice and services the veterinarians said they would offer and recommend to farmers included improvement in feeding, vaccination, use of artificial insemination, and adoption of farm biosecurity measures that can reduce disease prevalence, and improve food safety, animal health and welfare. Low use of personal protective equipment and other protective biosecurity measures among veterinarians could expose them to zoonotic diseases. The study concludes that there is a need for increased funding for continuous training, improved access to animal health-related information, and investment in infrastructure such as laboratories to enable veterinarians to deliver quality animal health services.

## Introduction

Veterinarians play an important role in the treatment and prevention of livestock diseases at the farm level (1). Moreover, they safeguard public health and serve as the first line of defence, preventing zoonotic diseases from spilling over into the human population (2). Many endemic cattle diseases are zoonoses and have a dual impact on both human health and livestock production in low-and middle-income countries (LMICs) such as countries in East Africa (3). Therefore, treating and preventing these diseases could alleviate poverty and reduce the health burden that disproportionately affects poor and marginalised populations (3, 4). Compared to developed countries, veterinary education, and animal health services in sub-Saharan Africa still lag behind in terms of human resources, funding and infrastructure (5, 6). Veterinarians in LMICs operate in resource-constrained environments that hinder their ability to contribute to the improvement of public health and food security (7). Although veterinarians in East Africa are resource-constrained, they play an important role in livestock production and safeguarding community livelihoods through the prevention and control of animal diseases (8).

Veterinary training curricula prepare veterinarians to recognise and initiate efficient animal disease control, apply effective treatment of diseased animals, enhance animal welfare, and safeguard human health (9). Veterinarians are trained and qualified to recognise livestock diseases through symptoms and laboratory diagnosis and to provide the correct treatment (1, 2). Although the number of veterinary schools and faculties has grown across Africa, leading to more veterinarians graduating and joining the workforce, the growth in student enrolment has not been matched with increased resources and infrastructure—such as enough teachers, classrooms and laboratories—which could compromise the quality of education and training (5, 10). Additionally, there is a need for continuous training of veterinarians on new emerging and re-emerging diseases and their new treatments as a way of improving the quality and preparedness of animal health services (4).

Veterinarians are themselves exposed to zoonotic diseases that can cause morbidity and mortality in their day-to-day occupational activities (11). However, veterinarians are trained and advised to strictly comply with biosecurity measures [also referred to as infection control practices (ICPs) which include the use of appropriate personal protective equipment (PPE) such as the use of gloves, masks gowns and boots] to protect themselves, their staff, and their clients, and to stop the spread of diseases or infections from one person, animal, or place, to others (12, 13). As there is a lack of studies investigating veterinarians’ adoption of biosecurity measures in LMICs, particularly in sub-Saharan Africa, this study aims to explore the use of ICPs by veterinarians in resource-poor settings (13).

There are numerous endemic livestock diseases in East Africa but access to quality veterinary services is a major challenge (14–16). Veterinarian-to-farmer ratios are low in these countries due to underfunding of veterinary services and privatisation necessitated by structural adjustment programmes of the early 1990s (7, 17). In East Africa, veterinarians are constrained by a lack of resources such as laboratory infrastructure and rely often on syndromic knowledge for disease diagnosis and treatment (17). This knowledge of typical symptoms characteristic of a specific disease has been developed through experience and years of practice treating endemic livestock diseases (18, 19). The knowledge is particularly crucial in resource-poor settings, such as in developing East African countries, where diagnostic laboratories are often absent (7, 19). Veterinarians play an important role in disease surveillance through their disease reporting and treatment of livestock at the farm level (7, 18, 19). However, the lack of resources hampers coordination between veterinary services and other relevant authorities leading to challenges in ensuring good disease management at the farm level and food safety from “stable to table” (8, 20). Furthermore, the lack of studies and evidence on the quality of veterinary services offered to farmers in East Africa hinders the improvement of veterinary services (21).

Improving animal health in East Africa could lead to the achievement of sustainable livelihoods and food security (3, 7, 17). Veterinarians could advise farmers regarding the on-farm adoption of biosecurity measures that can improve animal health, welfare and food safety (12). Additionally, veterinarians have an important role to play in the mitigation of antimicrobial resistance (AMR) by advising and encouraging farmers to adopt biosecurity and improve animal welfare, reducing infectious diseases, and antimicrobial stewardship (2, 22), the latter often referred to as an effort to measure and improve how antibiotic drugs are prescribed.

This study focuses on Ethiopia as a case study for several reasons. First, the country has one of the largest cattle populations in East Africa (23, 24). Therefore, it is urgent to prevent and treat livestock diseases to improve animal welfare and food safety and quality (4, 20, 24–29). Secondly, the availability, accessibility, and quality of animal health services is still a major challenge in Ethiopia (7, 19). The government-funded animal health systems that operate at local administrative levels known as “Kebele” are chronically underfunded and understaffed (19) These clinics lack laboratory and diagnostic facilities, and often the necessary medicines, to provide quality animal health services (7, 19). As veterinarians in the public animal health system often lack access to laboratory and diagnostic tools, this makes their syndromic knowledge of common diseases particularly important for disease diagnosis and treatment. However, the lack of supporting diagnostic tools has the potential to lead to wrong diagnosis and inappropriate use of antibiotics, which subsequently can contribute to the development of AMR among bacterial disease agents (19, 20, 22). Therefore, the main objective of this study was to explore veterinarians’ knowledge of common animal diseases, their treatment practices, the cost of treatment, their use of personal biosecurity measures, and their sources of information regarding diseases and treatment of animals in Ethiopia.

## Methodology

### Study area

The study was conducted between September and November 2021 in public and private veterinary clinic practices in Addis Ababa and its surrounding peri-urban areas (including Kaliti, Bole, and Kolfte), in Oromia (including Sendafa, Sebeta, Bishoftu, and Holeta towns), and around Adama town. The study areas were chosen for a number of reasons. First, they are important livestock production areas that supply milk and meat products to the fast-growing population. Second, livestock production is an important livelihood source, especially for smallholder farmers who dominate livestock production in the urban, peri-urban and rural areas in central Ethiopia (30, 31). A third reason was that livestock production by smallholder farmers faces the challenge of animal diseases endemic in Ethiopia (7). Finally, getting access to animal health services is a challenge for smallholder farmers in Ethiopia and particularly a problem in urban areas where urbanisation, climate change, and intensified livestock production are taking place (30–32).

### Questionnaire design

This study used a role-play approach to collect data. A survey questionnaire was designed based on an extensive literature review of the common cattle diseases in Ethiopia and the description of their most common symptoms (24, 27, 33–37). A rapid participatory rural appraisal (PRA) was conducted with veterinarians and researchers to identify cattle diseases of economic and public health importance. PRA involved visiting four animal health clinics in Addis Ababa and Oromia regions and Addis Ababa University College of Veterinary Sciences and discussing with veterinarians and researchers the important dairy cattle diseases in Ethiopia particularly in urban and peri-urban areas and the disease symptoms exhibited in sick cows.

The diseases selected for the survey (after the literature review and the PRA exercise) included brucellosis, bovine tuberculosis (bovine TB), mastitis, milk fever (calcium deficiency and hypocalcaemia), foot and mouth disease (FMD), lumpy skin disease (LSD), anthrax, swollen leg, abscesses and lameness, blackleg, fasciolosis and similar endoparasites, trypanosomiasis, and pasteurellosis. For each disease, a scenario was created that included a set of characteristic symptoms that are commonly manifested in a sick cow. In the questionnaire role-playing game, in each of the scenarios, the veterinarian was asked to treat the cases like a real livestock disease case and identify:

1. The disease.
2. What personal biosecurity measures they would take while examining the animal?
3. Whether they would conduct diagnostic tests before treatment.
4. What treatment they will recommend.
5. What would be the cost of treatment?
6. What additional services and inputs they would recommend to the farmer.
7. What would be the cost of the recommended inputs or services?
8. Whether they would report the disease to the authorities.
9. Their sources of information regarding diseases and treatment.

### Recruitment of veterinarians and questionnaire administration

Practising veterinarians were identified and sampled through judgmental non-probabilistic purposive and snowballing techniques due to a lack of information on the number of practicing veterinarians in the study area. The lead researcher relied on referrals from accessible practising veterinarians as getting government records was difficult, particularly personal information due to the civil war in the north which limited what could be shared by government institutions. The researchers therefore interviewed the veterinarians who were present and working in animal health clinics that were visited by the research team during the study period. Given the prevailing COVID-19 travel restrictions and war in Ethiopia, the researchers could only visit clinics in specific areas of Addis Ababa and Oromia.

The inclusion criteria were, (i) should be practising veterinarians either in public or private practice, (ii) willing to voluntarily participate in the study, and (iii) working on ruminants and other animals important for the food chain. Veterinarians were briefed on the study questionnaire, that their participation in the study was voluntary, and that confidentiality would be maintained before informed consent was obtained. The questionnaire was printed, and the veterinarians could fill in and add extra notes on the pages for each disease scenario. The questionnaire took 50–70 min to complete. The research had Ethical clearance from the University College London Research Ethics Committee (UCL-REC) approval number 19867/001 and the Armauer Hansen Research Institute (AHRI) and ALERT hospital AHRI/ALERT Ethics Review Committee (AAERC) approval (Protocol number PO-(46/14)).

### Participant observation and informal discussions data collection

Additionally, participant observation and informal discussions were conducted in six animal clinics in Addis Ababa, Adama and Oromia region at the kebele level to observe the issues covered in the questionnaire including the use of PPE, use of ICPs such as segregation of sick animals, the availability of diagnostic equipment and laboratories and treatment of livestock. Participant observations provided additional information allowed for data triangulation and eliminated the bias associated with self-reporting. These participant observations were recorded pictures by the lead author with prior consent from the veterinarians and also summarised as field notes. In each of the clinics, informal discussions were undertaken with one or two veterinarians in the animal clinics and covered issues around staffing, funding, availability of diagnostic laboratories, availability of PPE, quarantine and segregation facilities, and availability of drugs and livestock. In total eight veterinarians were engaged in the informal discussions. These informal discussions were recorded by the lead author with the consent of the veterinarians and were also summarised as field notes.

### Data management and analysis

The questionnaire data were entered into an Excel sheet and cleaned. The data were analysed for descriptive statistics including means and proportions using R statistical software. Tests were also undertaken to check for differences based on age, gender or year of graduation.

Thematic content analysis was undertaken on the informal discussions’ transcripts and the fieldnotes data. The analysis involved first familiarising with the data through an initial reading of the transcripts. Subsequently, the data was coded into major themes and then grouped into categories of similar ideas. Finally, ad-verbatim quotes were selected to contextualise the major findings.

Finally, the statistical results were compared with the informal discussions, participant observations and pictures collected to check for consistency and also to minimise self-reporting bias, e.g., people reporting they used PPE while that may not be true from participant observations.

## Results

### Demographic characteristics of respondents

Table 1 provides the demographic characteristics of the veterinarians who participated in the study. In total 48 veterinarians took part in the questionnaire survey; 47 had Doctor of Veterinary Medicine (DVM) or BSc in Veterinary Science degrees, while one had a BSc in Animal Health degree. The majority of the respondents were male and worked in the government-funded sector. The age profiles of both men and female veterinarians were similar and the majority of the participants graduated after the year 2000.

**Table 1:** Demographic characteristics of the participant veterinarians (*n* = 48).

		% (*n*)
Gender	Female	19 (9)
	Male	81 (39)
Mean age (in years)	Both genders	35 ± 10 years
	Female	34 ± 9 years
	Male	36 ± 10 years
Type of employment	Public veterinary clinic	95.8 (46)
	Private veterinary practice	4.2 (2)
Veterinary experience (in years)	Both genders	12 ± 10 years
	Female	11 ± 8 years
	Male	12 ± 11 years
University graduation year	1970s	8.3 (4)
	1980s	4.2 (2)
	1990s	12.5 (6)
	2000s	39.6 (19)
	2010s	35.4 (17)

### Syndromic cattle diseases knowledge

Table 2 summarises the veterinarians’ knowledge regarding 12 major cattle diseases in Ethiopia. There were no significant differences within the group based on age, gender or year of graduation. The majority of veterinarians correctly identified eight of the 12 different cattle disease scenarios based on the provided characteristic symptoms. However, reversely, only a minority was able to correctly identify pasteurellosis and trypanosomiasis, two diseases which are not common in the highlands of central Ethiopia. Instead, those who did not know frequently reported pasteurellosis as contagious bovine pleuropneumonia (CBPP) and trypanosomiasis as malignant catarrhal fever.

**Table 2:** Knowledge of diseases, notification, testing and treatment practices (*N* = 48).

Disease	Correct disease identification % (*n*)	Would notify about the disease % (*n*)	Would treat against the disease % (*n*)	Test before treatment % (*n*)
Brucellosis	100 (48)	33.3 (16)	66.7 (32)	33.3 (16)
Bovine tuberculosis (TB)	79.2 (38)	39.6 (19)	62.5 (30)	29.2 (14)
Mastitis	97.9 (47)	35.4 (17)	97.9 (47)	47.9 (23)
Milk fever (hypocalcaemia)	87.5 (42)	33.3 (16)	95.8 (46)	2.1 (1)
Foot and mouth disease (FMD)	97.9 (47)	18.8 (9)	75.0 (36)	14.6 (7)
Lumpy skin disease (LSD)	81.3 (39)	60.4 (29)	85.4 (41)	8.3 (4)
Anthrax	100 (48)	64.6 (31)	4.2 (2)	–
Swollen legs, abscesses, and lameness	45.8 (22)	70.8 (34)	87.2 (41)	2.1 (1)
Blackleg	68.8 (33)	33.3 (16)	100 (48)	16.7 (8)
Fasciolosis and other endoparasites	85.4 (41)	45.8 (22)	95.8 (46)	58.3 (28)
Trypanosomiasis	20.8 (10)	18.8 (9)	87.2 (41)	18.8 (9)
Pasteurellosis	18.8 (9)	39.6 (19)	87.2 (41)	14.6 (7)

The number of veterinarians who were willing to notify authorities about the notifiable diseases was low with less than 50% of veterinarians ready to notify most diseases, except for anthrax and LSD and swollen legs and abscesses, where 65% (*n* = 31), 60% (*n* = 29) and 71% (*n* = 34) would report, respectively, (Table 2). Veterinarians reported that it was a mandatory requirement to file weekly reports on all the cases, they had dealt with at the animal clinic. Livestock reporting was a requirement for an animal health information system which is meant to ensure Ethiopia has the surveillance capacity to monitor and control important livestock diseases.

In the case of anthrax, the majority of veterinarians recommended that the carcass of the dead animal should not be opened. They also suggested that such carcasses should be handled with appropriate personal protective equipment (PPE) (for example use of gloves, masks gowns and boots) and it should be burned, and the affected cattle shed disinfected. However, two participants suggested that live cattle suffering from anthrax diagnosed through symptoms should be treated with penicillin or other antibiotics. There was a consensus that sick animals should be isolated from healthy ones. In the case of bovine TB, although the majority said they would offer sick cattle antibiotics, only four suggested they would advise the farmers to not consume the milk or meat from the TB-infected cattle. The majority of the veterinarians (70.8%) suggested that they would advise the farmer to isolate bovine TB-infected cattle and/or cull them due to food safety risks associated with meat and milk consumption and the risk of disease transmission to healthy cattle.

### Treatments

Table 3 shows the various treatment options mentioned by the veterinarians for the treatment of the diseases, compared to the recommendations of the World Organisation for Animal Health: Home – WOAH (formerly, Office International des Epizooties-OIE). The majority of veterinarians recommended using antibiotics for the majority of the diseases.

**Table 3:** Treatments listed by the veterinarians for specific diseases, treatment duration cost mentioned by veterinarians compared to recommended management (*n* = 48).

Disease	Vets who said they would treat % (*n*)	Treatment and drugs reported by veterinarians	Treatment duration	Cost of treatment (Range, in ETB*)	Main recommendations stated by WOAH* and standard veterinary treatment guidelines of the drug administration and control authority of Ethiopia
Brucellosis	67.0 (32)	Antibiotics; Penstrip, Erythromycin, Oxytetracycline, Doxycycline, Streptomycin, Penicillin, Uterine bolus	3–7 days	300–1,500	Drug treatment: Chlortetracycline 6 to 10 mg/kg, intramuscular; other gram-negative susceptible drugs Treatment is not economical except in especially valuable animals, and even if the infection is eliminated, fertility may remain impaired Prevention: Test animals before introduction to a flock, cull infected animals, give awareness to the owner not to come into contact with foetal material and disinfect contaminated animals and surfaces
Bovine tuberculosis	63.0 (30)	Antibiotics; Tylosin, Penstrip, Isoniazid, Penicillin, Amoxicillin injection	3–7 days	200–1,500	Drug treatment: Treatment of bovine tuberculosis is not recommended because it is not economical Control and prevention: Tuberculin testing followed by segregation or culling of infected animals
Mastitis	98.0 (47)	Antibiotics, Tetracycline, Oxytetracycline, Penicillin, Tylosin, Penstrip and intramammary infusion	3–5 days	200–1,200	Clinical diagnosis by observing teat, udder and milk texture Treatment: intramammary infusion of Benzathine Cloxacillin, 500 mg for 3 days, Erythromycin 300 mg/quarter intramammary for 3–5 days Intramuscular injection of C/I, D/F, Streptomycin plus penicillin for 3–5 days
Milk fever (hypocalcaemia)	95.8 (46)	Calcium borogluconate, Calcium injection, Glucose	1–2 days	300–4,000	A typical treatment for an adult lactating dairy cow with periparturient hypocalcemia is 500 mL of 23% calcium borogluconate by slow intravenous injection. Intravenous administration is continued until the first arrhythmia is detected (a bradyarrhythmia such as a prolonged pause); the rate of intravenous administration is then slowed until a second arrhythmia is detected, at which time intravenous administration is discontinued and the remainder of the solution is placed subcutaneously over the lateral thorax
Foot and mouth disease (FMD)	75.0 (36)	Antibiotics, Penstrip, Sulphonamide, Oxytetracycline, Mild disinfectants (on wounds)	3–7 days	100–500	No specific treatment, however, supportive treatments against secondary bacterial infection are necessary Control and prophylaxis: Vaccination, test and quarantine of infected herds
Lumpy skin disease (LSD)	85.4 (41)	Antibiotics, Oxytetracycline, Penstrip	3–5 days	50–600	There is no effective treatment, but secondary bacterial infections are prevented by the administration of broad-spectrum antibiotics Prevention: Vaccination with sheep/goat poxvirus or LSD strain
Anthrax	0.0 (0)	–	–	200–2000	Drug treatment: Penicillin 22,000 IU/kg, IM, q 12 h for 2 days, then daily for 3 days or Benzathine penicillin or other repository preparations, q 48–72 h; the initial dose should be administered IV Prevention: Vaccination and animals that have died of anthrax should be burned in a closed incinerator or buried in the hole, to prevent animals not to access where animals had died. Anthrax is highly pathogenic to humans; thus, care should be taken during the handling of suspected cases
Swollen legs, abscesses and lameness	85.4 (41)	Antibiotics, Tincture of iodine, Procaine penicillin, Oxytetracycline, Penstrip, Drainage and removal of affected tissue	3–5 days	100–700	A superficial abscess may be treated by incision and drainage. Cleaning with hydrogen peroxide and iodine tincture. Isolating cattle to a housing with a smooth floor Prevention: improving the dairy house floor and applying rubber mats for the animal to lie on
Blackleg	100.0 (48)	Antibiotics, Atropine sulphate, Oxytetracycline, Penstrip, Penicillin	3–7 days	75–800	Drug treatment: Procaine penicillin G, 22,000 IU/kg, IM or SC q 24 h for 3 to 5 days or Benzathine penicillin or similar repository preparations, q 48–72 h. For S/E, C/I, D/F, D/I Prevention: Vaccination
Fasciolosis and other endoparasites	95.8 (46)	Anthelmintic drugs, Oxytetracycline, Triclabendazole, Fasionex, Penstrip, Albendazole, Bolus, Diminazine, Forsirex, Tefnocattle, Bensinedazolex	1 day and regular deworming	30–150	Treatment drugs are: Triclabendazole 9–12 mg/kg, PO stat (all stages of Fasciola). Albendazole 10 mg/kg, PO, start for mature stage Other endoparasites: anthelmintic bolus and ivermectin subcutaneous injection Control and prevention: regular seasonal deworming of animals
Trypanosomiasis	85.4 (41)	Antibiotics, Oxytetracycline, Penstrip, Topical repellent (Diminazine), Gentamycin, Diminazine aceturate (Berenil), Topical Gentamicin, Lidocaine	3–5 days	30–5,000	Drug treatment: Diminazene aceturate, Ethidium bromide and others Control: Control of tsetse flies includes frequent spraying and dipping of animals (mobile targets)., spraying insecticides on fly-breeding areas, bush clearing and other methods, insecticides-impregnated screens (fixed targets) and spray mobile target (e.g., Pour-on on cattle)
Pasteurellosis	85.4 (41)	Antibiotics, Oxytetracycline, Penstrip, Sulfadimidine, Oxytetracycline, Tylosin	3–5 days	50–2000	Drug treatment: Sulphadimidine 33%, IV, q 24 h for 3–5 days, Oxytetracycline 5–10 mg/kg IM or IV, q 12–24 h; Long-acting 20 mg/kg SC, IM or IV, q 2–4 days, -Penicillin–streptomycin 200,000 IU + 250 mg, 1 mL/25 kg Prevention: reduce animal stress and give a prophylaxis dose of Oxytetracycline long-acting 20 mg/Kg, IM

*ETB, Ethiopian birr; 1 US dollar is equivalent to 51 Ethiopian Birr (29th June 2022); *WOAH, The World Organisation for Animal Health, an intergovernmental organization coordinating, supporting, and promoting animal disease control; AI, artificial insemination.

### Other recommendations and animal health advice to farmers

Table 4 presents the additional services, inputs, and advice that participating veterinarians said they would recommend to farmers. The veterinarians said they would provide advice and recommend measures on food safety, biosecurity measures, animal welfare, livestock feeding and farm sanitation and hygiene. These recommendations included also inputs such as vitamins, deworming, vaccinations, and artificial inseminations (AI). The cost of these inputs was rated at 100–550 ETB for multivitamins, 100–2000 ETB for different vaccinations, and 200–500 ETB for AI.

**Table 4:** Veterinarians’ recommended management practices for the diseases.

Disease	Other recommendations given by veterinarians
Brucellosis	Vitamins, deworming, uterine bolus, giving a balanced diet, vaccinating, disinfecting the area where a cow aborts, handling aborted foetus aseptically, preventing contact with other herds, culling of affected cow, separating animals (isolation), proper disposal of the aborted foetus and the placenta, use of artificial insemination (AI)
Bovine tuberculosis	Deworming, multivitamins, giving a balanced diet, advice not to consume meat/milk, isolating the sick animal from the herd, culling the affected animal, advising to boil milk before consumption, advising on economic and public health significance, testing and culling
Mastitis	Multivitamins, improve shed cleanliness, improve milking and farm hygiene, sanitize cow udders, proper farm sanitation, disinfect the milking area, teat dipping, teat sealing, good hygienic practices, intramammary antimicrobial treatment, isolate sick cows, milk sick cows last, provide clean sleeping areas
Milk fever (hypocalcaemia)	Multivitamins, food supply with calcium content, steroids, providing the animal with well-balanced feeds, maintaining calcium in feeds, supplementing with calcium for 2–3 weeks pre-calving
Foot and mouth disease (FMD)	Multivitamins, control animal movement, limit contact with cows, annual vaccination, isolate the sick animals, proper wound management in sick animals, quarantine, washing the wound with salts, avoiding the use of community pasture, cleaning the barn regularly, and tick control
Lumpy skin disease (LSD)	Multivitamins, vaccination annually, isolation of sick cows, and good biosecurity management
Anthrax	Cull affected animals, vaccinate healthy animals, educate farmers on the handling of infected bodies, dispose of dead cows by burning, provide antibiotics early to infected cows, bury dead animals, and disinfect cattle sheds
Swollen legs, abscesses and lameness	Multivitamins, tincture of iodine, use of antibiotics, cattle vaccination, drainage and removal of affected tissue, deworming, improving cattle housing, separating/isolating and caring for sick animals, improving farm hygiene, cleaning barn regularly, keeping shed floors dry, culling chronically sick cows
Blackleg	Multivitamins, tetanus antitoxin, deworming, isolation of sick cows, increase farm biosecurity, annual vaccination, proper disposal of the carcass, preventing the animals from contact with other herds, advice on completing the dose
Fasciolosis and other endoparasites	Multivitamins, regular deworming, avoiding marshy areas for grazing, clean drinking water to be given to the animal, do not consume meat or milk from treated cows, balanced feed and nutrition
Trypanosomiasis	Multivitamins, annual vaccination, isolating sick animals, cleaning the house frequently, avoiding the use of deltamethrin, deworming, not buying an animal from trypanosomiasis affected area, monitoring drug resistance, and tick control
Pasteurellosis	Multivitamins, isolate sick animals, cull sick animals, early treatment of affected cows, have good biosecurity management, and clean and air cattle housing

### Adoption of biosecurity measures by veterinarians

Table 5 presents the personal biosecurity measures that would be adopted by veterinarians while handling cattle sick with various diseases. There were no significant differences within the group based on age, gender or year of graduation. There was low use of PPE by veterinarians to protect themselves against occupational risks, particularly notable in the case of zoonotic diseases such as bovine TB, brucellosis, and anthrax. Only 55% of the participating veterinarians were washing their hands after contact with sick cattle. Among the veterinarians, only 50% used gloves, gowns and overalls. All the veterinary clinics that were visited for participant observations lacked water and handwashing facilities, and the laboratories boiled their equipment in hot water or a pressure cooker for disinfection. Apart from some light microscopes, the majority of the clinics lacked a functioning laboratory for disease diagnosis. Instead, animal clinics referred animal tissue samples to national laboratories for analysis if they suspected an important livestock disease of economic or public health importance.

**Table 5:** The reported personal protection equipment and infection control practices veterinarians would use in disease scenarios (*n* = 48).

	None	Hand wash after contact	Gloves only	Gown/overalls only	Gloves & gown/overalls	Gloves, gown/overalls & face protection (respiratory mask and goggles)	Gloves, overalls with head protection, P2 respiratory mask and goggles	Not sure
Brucellosis	0.0 (0)	54.2 (26)	10.4 (5)	16.7 (8)	29.2 (14)	37.5 (18)	10.4 (5)	2.1 (1)
Bovine tuberculosis	0.0 (0)	50.0 (24)	4.2 (2)	6.3 (3)	31.3 (15)	39.6 (19)	8.3 (4)	14.6 (7)
Mastitis	4.2 (2)	50.0 (24)	8.3 (4)	8.3 (4)	41.7 (20)	14.6 (7)	2.1 (1)	18.8 (9)
Milk fever (calcium deficiency)	8.3 (4)	41.7 (20)	6.3 (3)	8.3 (4)	41.7 (20)	18.8 (9)	0.0 (0)	2.1 (1)
Foot and mouth disease (FMD)	10.4 (5)	56.3 (27)	6.3 (3)	8.3 (4)	35.4 (17)	29.2 (14)	8.3 (4)	6.3 (3)
Lumpy skin disease (LSD)	2.1 (1)	43.8 (21)	10.4 (5)	6.3 (3)	50.0 (24)	22.9 (11)	6.3 (3)	14.6 (7)
Anthrax	18.8 (9)	43.8 (21)	2.1 (1)	4.2 (2)	27.1 (13)	27.1 (13)	25 (12)	6.3 (3)
Swollen legs, abscesses and lameness	18.8 (9)	41.7 (20)	8.3 (4)	10.4 (5)	41.7 (20)	16.7 (8)	2.1 (1)	4.2 (2)
Blackleg	16.7 (8)	43.8 (21)	4.2 (2)	14.6 (7)	58.3 (28)	6.3 (3)	4.2 (2)	0.0 (0)
Fasciolosis and other endoparasites	14.6 (7)	52.1 (25)	6.3 (3)	8.3 (4)	43.8 (21)	8.3 (4)	0.0 (0)	0.0 (0)
Trypanosomiasis	14.6 (7)	50.0 (24)	8.3 (4)	4.2 (2)	37.5 (18)	27.1 (13)	0.0 (0)	0.0 (0)
Pasteurellosis	18.8 (9)	37.5 (18)	2.1 (1)	6.3 (3)	41.7 (20)	14.6 (7)	8.3 (4)	4.2 (2)

Results in % (*n*).

### Information sources for veterinarians in Ethiopia

The majority of veterinarians (83.3%) reported that their knowledge regarding animal diseases and treatment was a result of experience working with livestock. Additionally, this knowledge regarding animal diseases and treatment was disseminated through social networks within the veterinary community. Veterinarians also relied on farmers, and colleagues working as animal extension officers as a trusted source of information. They also reported using University training notes handouts (notes used by lecturers to train students), The Merck Veterinary Manual (MSD veterinary manuals), veterinary books, the internet (veterinary websites), standard treatment guidelines (provided by drug manufacturers), and standard veterinary guidelines/manuals as a source of information regarding emerging diseases and treatment and new developments within the wider veterinary profession.

### Participant observation and informal discussion results

Participant observations and informal discussions with veterinarians in this study revealed low use of PPE, with the majority of veterinarians using only lab coats and gloves when treating livestock or doing reproductive canal examinations.

“*There is no supply* [of] *gloves, syringes and other PPE, so this is a big challenge for us and hampers provision of a good quality animal health service* [to farmers].” Veterinarian 4, Clinic C, Oromia.

“*As you can see in our clinic, we do not have a clinical room or space to separate risk cases. We are trying as much as possible to protect ourselves, but there is a gap* [on use of PPE]” Veterinarian 2, Clinic B, Addis Ababa.

Moreover, the clinics posed animal-to-human and animal-to-animal disease transmission risks, as sick livestock from different farms were allowed to mix at the clinics’ compounds since they lacked isolation and quarantine sheds or holding areas. Additionally, animal clinics were not regularly disinfected or cleaned to reduce livestock disease transmission risks. Moreover, some clinics lacked the necessary facilities to work in or store medicine.

“*I have been using* [disposable plastic shopping] *bags to cover my shoes when visiting farms, to avoid transmission of contagious diseases like FMD. Only a handful of farms have a foot bath with “berakina”* [disinfectant].” Veterinarian 3, Clinic C, Addis Ababa.

In all the visited clinics there were poor routines for disposal of medical waste, particularly drug vials and gloves which were often littered in the compound of the clinics. Furthermore, all the clinics lacked incinerators to dispose of infectious materials and/or animal tissues which could increase the risks of pathogen transmission within the clinics and also risk environmental contamination.

Six of the eight veterinarians engaged during the informal discussions revealed that animal health clinics often lacked drugs and that purchasing the drugs was costly, particularly for smallholder farmers in rural areas. Quality drugs were perceived as expensive and inaccessible in rural areas. The majority of veterinarians (seven out of the eight) thought that irrational drug prescription and abuse by farmers and veterinarians were the cause of drug-resistant disease agents, and they reported that they were experiencing resistant infections which required the use of stronger antibiotics. However, despite the scarcity of drugs, the negative attitude and behaviour of farmers including imprudent use of antibiotics purchased over-the-counter drugs to treat livestock without consulting a qualified veterinarian were seen as a challenge by the veterinarians. Four of the eight veterinarians believed that over-the-counter medicines were leading to pathogen resistance and making treatment ineffective. Due to their reliance on syndromic knowledge rather than laboratory disease diagnostics, veterinarians perceived farmers as having poor skills in explaining cattle disease case history which led to insufficient information for correct disease diagnosis and treatment.

“There is a drug supply problem and farmers complain when we cannot provide them.” Veterinarian 2, Clinic B, Oromia.

Finally, three veterinarians reported that climate change was causing the emergence of new diseases and the re-emergence of endemic diseases. Cattle diseases harm livestock production and farmers’ livelihoods. They also reported that seasonal feed availability and shortages were impacting livestock health and that poor nutrition meant that the cattle had low immunity to cope or fend off diseases.

### Challenges faced by veterinarians in Ethiopia

The majority of the veterinarians in this survey (83.3%) reported that the lack of laboratory equipment, reagents, and material for the diagnosis of animal diseases was a major challenge that hampered their service delivery.

“[the budget] *is below sufficient* […] *Firstly, we cannot give enough vaccines. There is a lack of enough quantity of medication here. That affects us in choosing the drug for treatment. Low availability of these medications hampers service delivery*” Veterinarian 1, clinic A, Addis Ababa.

There is a lack of accessible laboratories for disease diagnosis, particularly in government-funded clinics in Ethiopia. Veterinarians reported that their laboratory diagnosis and clinical skills were below the expected laboratory standards and norms and that there was a need for regular refresher training on disease diagnosis and treatment. Moreover, veterinarians also reported the lack of PPE which exposes them to zoonoses and other occupational risks. Finally, veterinarians cited that the lack of PPE was because of low budget allocation and a long bureaucratic procurement process.

“*What I believe is lacking is supporting laboratory examinations to inform treatment. That is due to manpower shortage and absence of laboratory*” Veterinarian 1, clinic A, Addis Ababa.

“*As you can see there is no assigned lab professional. We have a microscope and do sometimes faecal and blood samples. But it is not fully functional*.” Veterinarian 6, Clinic C, Oromia.

## Discussion

The main objective of this study was to explore veterinarians’ syndromic knowledge of the common diseases they are facing, their treatment practices, the cost of treatment, their use of personal biosecurity measures, and their sources of information regarding diseases and treatment in Ethiopia. Previous studies have documented that livestock diseases, particularly cattle diseases, are a priority problem for farmers throughout Ethiopia (4, 25). Farmers have singled out poor animal health service delivery as the major constraint to improving animal health and productivity (10, 25). The findings of this study show that the majority of the veterinary practitioners had good knowledge of the most common endemic cattle diseases in Ethiopia, however, a knowledge gap was seen for cattle diseases that are not common in the highlands of Ethiopia, such as pasteurellosis and trypanosomiasis. The majority of veterinarians reported that they were likely to not use PPE when treating cattle diseases even though some of these diseases were zoonotic. The practice of treating diseased animals with antibiotics without confirmed laboratory diagnosis could lead to wrong treatments and an increased risk of circulating pathogens becoming drug-resistant. Furthermore, there were differences between the reported treatments and the standard recommended treatments by the World Organisation for Animal Health (WOAH) and the drug administration and control authority of Ethiopia guidelines.

The majority of participants in this study were male and working in government-funded animal clinics. Previous studies have reported a low number of women veterinarians in the wider East Africa, which calls for policies that can lead to retention and employment of more women in animal health services (10). Although private practices were not explicitly investigated in this study, previous studies have also reported that private veterinary practices, other than in South Africa, are very limited and under-developed, which has led to farmers’ dependence on government-funded animal health services (7, 19). There is a need to increase gender diversity and the plurality of service providers and this can be achieved with the growth in veterinary medical schools in Ethiopia (5, 7, 10). Previous studies have reported that veterinary training in Africa for many years has focused on producing veterinarians to work for the state in public animal health services in the livestock sector (7). Furthermore, the human resource gap in veterinary sectors in low-income countries, particularly in sub-Saharan Africa, imposes limitations on the delivery of animal health services (38).

Veterinarians depend on their training, experience and social networks for communication regarding diseases and treatment (1, 5, 9, 10). The majority of the veterinary practitioners had good knowledge of most of the endemic cattle diseases in Ethiopia but failed to a large extent to recognise diseases such as pasteurellosis and trypanosomiasis which are not common in the highlands of Ethiopia. However, these and other diseases could in the future become more prevalent in the highlands due to climate change, globalisation, and the introduction of new cattle breeds susceptible to endemic zoonoses (4). The case descriptions were only of a selected number of diseases, using typical pathognomonic symptoms, and the study did not evaluate how vaguer symptoms would have been diagnosed, such as a case of only abortions, which could be due not only to brucella but also to some other diseases, including those with bacterial and viral origin (39).

The results reveal low disease reporting and notification although it is a mandatory requirement to file weekly reports on cases attended at the animal clinic. Low notification of diseases hampers disease surveillance which could be detrimental to animal health management (4, 22, 39). Previous studies have reported the underfunding of veterinary services in sub-Saharan Africa that hampers animal health service delivery (4, 19). There is a need for animal health services investments as envisioned in the “One-health approach” given the numerous endemic livestock diseases and zoonoses in sub-Saharan Africa (4).

The results of this study show that the recommended treatment suggested to farmers occasionally was not in line with the recommendations of the WOAH and the guidelines from the drug administration and control authority of Ethiopia (Tables 3, 4). For the majority of diseases, the surveyed veterinarians could propose treatment methods which they reported were based on their experience and information from their social networks. The choice of antibiotics used by veterinarians in this study is similar to what has been reported in previous studies in sub-Saharan Africa (40) and also in India (41). Moreover, there is a need to ensure that farmers have access to qualified veterinarians to get information and advice on the prudent use of antibiotics particularly concerning the use of over-the-counter drugs to treat livestock which creates the risk for AMR (22, 42, 43). Moreover, the practice of using antibiotics for prophylaxis contravenes antimicrobial stewardship and could exacerbate the challenges posed by the development of AMR pathogens (10, 22, 41). Previous studies have shown that the indiscriminate use or misuse of antibiotics by veterinarians and farmers for animal treatment contributes to AMR problems (10, 22). It is thus imperative for training and awareness creation on the risk of AMR and the need for antibiotic stewardship (40, 41).

The extra services and advice recommended by veterinarians to farmers in this study (Table 4), such as improvement in animal feeding, vaccination, use of AI and adoption of farm biosecurity measures, can reduce disease prevalence, and improve food safety, animal health and welfare (7, 12). Veterinarians are professionals in close contact with farmers and thus are trusted sources of information (44, 45). Adoption of farm biosecurity measures and improved farm hygiene practices as recommended in the findings of this study has the potential to reduce livestock disease prevalence at the farm and lead to improved food safety for consumers (8, 12).

Veterinarians working in government-funded animal clinics faced challenges such as lack of PPE, underfunding of the animal health services budget and low supply of essential medicines and pieces of equipment. There was a low reported use of PPE, a behaviour that exposes veterinarians to occupational risks including exposure to deadly zoonoses such as anthrax, bovine TB, and brucellosis. There is a need to increase the use of PPE to reduce the risk of zoonotic diseases in their day-to-day occupational activities (11). Moreover, the poor disposal of medical and animal tissues at animal clinics could contaminate the environment and lead to the transmission of livestock diseases, particularly those that can persist in the soil and groundwater, such as anthrax and Q-fever (7, 46).

The majority of veterinarians obtained information on animal diseases and drugs from their social networks, training manuals and their lived experience. Previous studies have reported the importance of experience and syndromic knowledge for disease diagnosis and treatment (7, 47). There is a lack of a centralised information system in the Ethiopian veterinary sector which constrains the quality of animal health services (7). Similar to the findings of this study, Gizaw et al. (25) recommended the establishment of an integrated animal health information system to improve veterinary service delivery in Ethiopia. The use of new media technologies can help bridge the information gap while at the same time providing new avenues to learn new skills and technologies (48, 49).

The adoption of new and emerging technologies can provide cheap and innovative solutions that can address the lack of diagnostic facilities (49, 50). The lack of laboratory facilities as evidenced in this study can be addressed through new emerging technologies such as mobile diagnostic units, digital technologies, rapid test kits and telemedicine which can improve disease surveillance and diagnosis capabilities in remote and resource-poor settings (50, 51). However, to leverage these new emerging technologies, there is a need for private and public sector investments in procuring rapid test kits, training on how to use the new technologies, and expanding access to the internet (49, 50). Increasing access to animal health information such as animal disease outbreaks is crucial to safeguarding animal, human and environmental health and livelihoods (7, 10). Although beyond the scope of this study, there are opportunities to leverage community animal health workers and extension workers to advise farmers and provide routine services to livestock farmers which can improve access to animal health services (52, 53). Moreover, adopting new and innovative technologies can contribute to the retention of women veterinarians in the workforce in emerging economies such as Ethiopia by removing barriers that make women leave veterinary occupation such as salary, work-family balance, working hours, and workload (54, 55). Expanding women and youth participation in animal health services can create more resilient and responsive veterinary health services that reflect local needs and contextual realities (55–57).

The findings of this study show that veterinarians rely on experience and syndromic knowledge to provide animal health services due to a lack of diagnostic facilities. There is therefore a need for regular training to ensure that veterinarians are aware of new emerging zoonoses and re-emerging zoonoses (7, 10). Training veterinarians on interdisciplinary thinking and collaborative working such as the implementation of One Health-focused training can harness existing knowledge and resources to improve health human, animal and environmental health (29). Global and regional initiatives such as Africa One Health University Network (AFROHUN), the International Livestock Research Institute (ILRI), the Food and Agriculture Organisation of the United Nations (FAO), and the One Health Research, Education and Outreach Centre in Africa (OHRECA) project are working to transform the training and capacity building in Africa which could benefit veterinarians working in resource-poor setting (58, 59).

## Limitations

This study looked to explore how livestock treatment occurs in a real-world environment in Ethiopia and how veterinarians diagnose and treat cattle diseases. In this study, we did not have the exact number of practising veterinarians in the areas we were undertaking research and as such we relied on convenience and purpose sampling to reach as many respondents as possible. Moreover, due to COVID-19 and the civil war in Ethiopia, the lead researcher’s travel plans were restricted to the relatively safe areas permitted by the Ethiopian government which made it impossible to reach as many veterinarians as we could have wanted which could have created a selection bias towards veterinarians who were easily accessible under the prevailing circumstances at the time.

Although this study provides insight into animal health services in Ethiopia, the sample size was too small to generalise regarding trends across the country. We believe our choice of mixed method approach involving qualitative and quantitative data provides an insight into the general trends across the country. Future studies should therefore aim for bigger sample sizes, and reach a diverse set of animal health practitioners (e.g., veterinarians, paravets, community animal health workers). Moreover, experiments and longitudinal research approaches could also explore the causality between the quality of animal health services and animal health outcomes. Future studies should also explore how new modern technologies such as digital technologies and rapid test kits can improve animal health services and also improve animal disease monitoring and surveillance.

Finally, due to budget limitations, this observational study was not set to compare syndromic knowledge and clinical diagnosis of disease through laboratory analysis. Future studies should compare the accuracy of syndromic knowledge to laboratory analyses to explore ways to help veterinarians in their disease assessments given that most livestock treatment still depends on experience and syndromic knowledge. There is a need to ensure that correct disease diagnoses are made before administration of treatment to mitigate and avoid AMR.

## Conclusions and policy implications

Poor quality of animal health services has been documented in Ethiopia. There is an urgency to improve the coverage and quality of veterinary services across Ethiopia to improve productivity, animal welfare and public health. Veterinarians play an important role in preventing and treating livestock diseases and also safeguarding human health. However, their capacity to offer quality services is constrained by underfunding and the absence of infrastructure such as the lack of laboratories, PPE, understaffing and underfunding of animal health services constrain livestock production. Policymakers should prioritise the establishment of a national veterinary health information system for animal disease monitoring and surveillance. Moreover, there is a need for policies that provide incentives for private sector involvement in veterinary services to bridge the animal health services coverage gap and particularly serve farmers who are willing to pay for animal health services.

There is a need for a concerted multi-sectoral approach to improve veterinary service delivery through improved veterinary infrastructure and public-private partnerships. The absence of diagnostic facilities and susceptibility tests hinders the prescriptions of the right antimicrobial drugs, which could subsequently lead to increased AMR. Increased funding, continuous training, improved access to information and investment infrastructure such as laboratories would enable veterinarians to deliver quality animal health services to farmers. Additionally, there is a need to encourage veterinarians to adopt and use protective biosecurity measures to reduce their exposure to zoonoses.

## Data Availability

The raw data supporting the conclusions of this article will be made available by the authors, without undue reservation.
