# Supplementary material for: The role of syndromic knowledge in Ethiopian veterinarians’ treatment of cattle
Source: Front Vet Sci. 2024 Aug 30;11:1364963. doi: 10.3389/fvets.2024.1364963 (PMC11392921; doi:10.3389/fvets.2024.1364963)
Supplement: Supplementary file 1 [file Data_Sheet_1.pdf]

Cover page

Name (ID) __________________

Age: _________________________

Gender: ____________________________

Area/Clinic: ____________________________

Year of graduation ______

Specialisation: Farm animals _____ equine ______ pets ______ mixed ______

Type of employment: Private practice________ Public employees ________

How many years of practice? ____________________________________

Scenario 1

A farmer calls you about their cattle that show the following clinical signs. A cow has just aborted on the 7th month of pregnancy. After abortion, the afterbirth doesn't come out (retained placenta). The part of placenta exposed shows signs of infection (placentitis). The farmers explains that his cows have reduced their milk yield and a number of animals in the herd has had abortions before.

What is the likely disease?	
What treatment (drugs) will you recommend	
How long will the treatment last	
What else will you recommend for example vitamins, deworming	
Would submit samples before treatment?	
If yes what treatment will you administer as you wait for results	
Would you notify authorities about this case?	
What will be the total cost for treating the disease?	
What will be the total cost for recommended extra products/services?	
What else would you recommend to the farmer?	

What biosecurity will you take when examining and attending the cows

None	
Hand wash after contact	
Gloves only	
Gown/overalls only	
Gloves & gown/overalls	
Gloves, gown/overalls & face protection (respiratory mask and goggles)	
Gloves, overalls with head protection, P2 respiratory mask and goggles	
Not sure	

Any more comments:

Scenario 2

A farmer calls you about their cattle that show the following clinical signs. The cow has a and looks under distress. The animal has a persistent soft cough, a fluctuating fever. The farmer explains that the cow has shown progressive weight loss decreased appetite, looks weak and poor body score. The cow has impaired breathing (increased breathing rate and discharge of yellowish secretion from the nose). The Superficial lymph nodes are enlarged (swollen nodes). The animal has mastitis like symptoms including inflammation at the base of the quarter of the udder with painless swellings.

What is the likely disease?	
What treatment (drugs) will you recommend	
How long will the treatment last	
What else will you recommend for example vitamins, deworming	
Would submit samples before treatment?	
If yes what treatment will you administer as you wait for results	
Would you notify authorities about this case?	
What will be the total cost for treating the disease?	
What will be the total cost for recommended extra products/services?	
What else would you recommend to the farmer?	

What biosecurity will you take when examining and attending the cows

None	
Hand wash after contact	
Gloves only	
Gown/overalls only	
Gloves & gown/overalls	
Gloves, gown/overalls & face protection (respiratory mask and goggles)	
Gloves, overalls with head protection, P2 respiratory mask and goggles	
Not sure	

Any more comments:

Scenario 3

A farmer calls you about their cattle that show the following clinical signs. The cow has high fever, has reduced appetite and has reduced mobility/movement (look for right word). The cow udder is swollen. there is some hardness in the udders and the affected teat feels hot. Additionally, the udders show redness and the cow looks to be in pain. The farmer reports that since the problem started the cow milk flow and production has reduced significantly and one of the teats is blocked. Upon inspection you find that milk appears watery with small clot lumps. Observation reveals dirty cow shed with wet bedding.

What is the likely disease?	
What treatment (drugs) will you recommend	
How long will the treatment last	
What else will you recommend for example vitamins, deworming	
Would submit samples before treatment?	
If yes what treatment will you administer as you wait for results	
Would you notify authorities about this case?	
What will be the total cost for treating the disease?	
What will be the total cost for recommended extra products/services?	
What else would you recommend to the farmer?	

What biosecurity will you take when examining and attending the cows

None	
Hand wash after contact	
Gloves only	
Gown/overalls only	
Gloves & gown/overalls	
Gloves, gown/overalls & face protection (respiratory mask and goggles)	
Gloves, overalls with head protection, P2 respiratory mask and goggles	
Not sure	

Any more comments:

Scenario 4

A farmer calls you about their cattle that show the following clinical signs. The farmer reports that a pregnant cow that is nearly giving birth has fallen over and remains seated with its head resting on its shoulder and is shivering. Your examination reveals a slight drop in temperature. the farmer reports that during the week the animal has been dull and lost appetite.

What is the likely disease?	
What treatment (drugs) will you recommend	
How long will the treatment last	
What else will you recommend for example vitamins, deworming	
Would submit samples before treatment?	
If yes what treatment will you administer as you wait for results	
Would you notify authorities about this case?	
What will be the total cost for treating the disease?	
What will be the total cost for recommended extra products/services?	
What else would you recommend to the farmer?	

What biosecurity will you take when examining and attending the cows

None	
Hand wash after contact	
Gloves only	
Gown/overalls only	
Gloves & gown/overalls	
Gloves, gown/overalls & face protection (respiratory mask and goggles)	
Gloves, overalls with head protection, P2 respiratory mask and goggles	
Not sure	

Any more comments:

Scenario 5

A farmer calls you about their cattle that show the following clinical signs. The cows have fever and vesicles (blisters) chiefly in the mouth and on the muzzle, teats, and feet. Sick cows have a rough coat, have quivering lips and frothing of mouth, are stamping their feet when standing but most of the sick animals prefer to lie down. The cows have lost appetite and reduced feed intake. Milk production has gone down in the herd. A number of the pregnant cows have aborted. A number of calves have died from similar symptoms.

What is the likely disease?	
What treatment (drugs) will you recommend	
How long will the treatment last	
What else will you recommend for example vitamins, deworming	
Would submit samples before treatment?	
If yes what treatment will you administer as you wait for results	
Would you notify authorities about this case?	
What will be the total cost for treating the disease?	
What will be the total cost for recommended extra products/services?	
What else would you recommend to the farmer?	

What biosecurity will you take when examining and attending the cows

None	
Hand wash after contact	
Gloves only	
Gown/overalls only	
Gloves & gown/overalls	
Gloves, gown/overalls & face protection (respiratory mask and goggles)	
Gloves, overalls with head protection, P2 respiratory mask and goggles	
Not sure	

Any more comments:

Scenario 6

A farmer calls you about their cattle that show the following clinical signs. The cows have a fever that go up and down (goes down after 1 - 2 days but it goes up again) and are salivating profusely and are showing grayish/white discharge from the nose. The cows have swollen lumps on the body around the head and neck, under the abdomen, on the legs around the genitals and the udder which about the same size of a coin in diameter. The lumps are firm, raised, round and hair on the lumps stands up. The open lumps on the skin are peeling leaving open wounds. The sick cows show enlarged superficial lymph nodes. One of the pregnant cows has aborted. Milk production in lactating has also reduced. One of the calves has also died from the symptoms. The farmer reports that the sick cows are weak and have reduced feed intake.

What is the likely disease?	
What treatment (drugs) will you recommend	
How long will the treatment last	
What else will you recommend for example vitamins, deworming	
Would submit samples before treatment?	
If yes what treatment will you administer as you wait for results	
Would you notify authorities about this case?	
What will be the total cost for treating the disease?	
What will be the total cost for recommended extra products/services?	
What else would you recommend to the farmer?	

What biosecurity will you take when examining and attending the cows

None	
Hand wash after contact	
Gloves only	
Gown/overalls only	
Gloves & gown/overalls	
Gloves, gown/overalls & face protection (respiratory mask and goggles)	
Gloves, overalls with head protection, P2 respiratory mask and goggles	
Not sure	

Any more comments:

Scenario 7

A farmer calls you about their cattle that show the following clinical signs. The farmer reports of sudden death of cattle in the herd (within 2-3 hrs). The farmer reports the cattle was staggering, trembling, had a high temperature, difficulty breathing. The cattle collapsed and had convulsions before death.

You notice small amount of bloody discharge from the nose, mouth and other openings of the dead cow. And they want your advice, how to handle the dead body because they fear of last experience, in which the diseases transmitted to other animals by feeding on the area where dead body exposed. So what will be your advice them and action you take?

What is the likely disease?	
how will you handle the body?	
Would submit samples for analysis?	
Would you notify authorities about this case?	
What will be the total cost for disposing the body?	
What else would you recommend to the farmer?	

What biosecurity will you take when examining and attending the cows

None	
Hand wash after contact	
Gloves only	
Gown/overalls only	
Gloves & gown/overalls	
Gloves, gown/overalls & face protection (respiratory mask and goggles)	
Gloves, overalls with head protection, P2 respiratory mask and goggles	
Not sure	

Any more comments:

Scenario 8

A farmer calls you about their cattle that show the following clinical signs. a number of cows have swollen legs and signs of lameness. There are swelling in the hind legs and sores on the skin. The affected animals have difficulty standing and moving. The cows have sole bruising, toe abscess, sole abscess and abscess in the inter digital space.

The cow has severe lameness on the back legs and holds the leg in air as if to relieve pressure and the cow tends to stand and walk on their toes. Additionally, there is a swelling of interdigital space. The cow has fever, poor body score and has reduced milk yields. The affected animals are reluctant to travel to feed and water. foot is a sub-acute or acute necrotic infection originating from a lesion in the interdigital skin that leads to a cellulitis in the digital region.

What is the likely disease?	
What treatment (drugs) will you recommend	
How long will the treatment last	
What else will you recommend for example vitamins, deworming	
Would submit samples before treatment?	
If yes what treatment will you administer as you wait for results	
Would you notify authorities about this case?	
What will be the total cost for treating the disease?	
What will be the total cost for recommended extra products/services?	
What else would you recommend to the farmer?	

What biosecurity will you take when examining and attending the cows

None	
Hand wash after contact	
Gloves only	
Gown/overalls only	
Gloves & gown/overalls	
Gloves, gown/overalls & face protection (respiratory mask and goggles)	
Gloves, overalls with head protection, P2 respiratory mask and goggles	
Not sure	

Any more comments:

Scenario 9

The farmers report of sudden cow death in the herd. The deaths occur after 12–48 hours after the onset of symptoms. Additionally, there are animal showing similar symptoms in the herd. The farmers explains that the cow became suddenly lame with swelling of a muscles, stopped grazing, appeared sick and quickly went down. The cow had developed lesions without any history of wounds. Some animal show acute, severe lameness commonly affecting the hind legs. The affected cow temperature is sub-normal. The affected cows have swellings in the hip, shoulder, chest, back, neck, or elsewhere which are focal, hot, and painful. There is also grinding, creaking, cracking or popping that occurs when moving a join in the affected cows.

What is the likely disease?	
What treatment (drugs) will you recommend	
How long will the treatment last	
What else will you recommend for example vitamins, deworming	
Would submit samples before treatment?	
If yes what treatment will you administer as you wait for results	
Would you notify authorities about this case?	
What will be the total cost for treating the disease?	
What will be the total cost for recommended extra products/services?	
What else would you recommend to the farmer?	

What biosecurity will you take when examining and attending the cows

None	
Hand wash after contact	
Gloves only	
Gown/overalls only	
Gloves & gown/overalls	
Gloves, gown/overalls & face protection (respiratory mask and goggles)	
Gloves, overalls with head protection, P2 respiratory mask and goggles	
Not sure	

Any more comments:

Scenario 10

The owner complained about his animal shows a weight loss, anaemia, bottle jaw and diarrhoea. The cattle have reduced milk yield, depressed fertility, excessive weight loss and lower body condition scores. some cows that have given birth had lower birth weight of calves. Calves have a reduction in daily weight gain. The animal show also bottle jaw.

What is the likely disease?	
What treatment (drugs) will you recommend	
How long will the treatment last	
What else will you recommend for example vitamins, deworming	
Would submit samples before treatment?	
If yes what treatment will you administer as you wait for results	
Would you notify authorities about this case?	
What will be the total cost for treating the disease?	
What will be the total cost for recommended extra products/services?	
What else would you recommend to the farmer?	

What biosecurity will you take when examining and attending the cows

None	
Hand wash after contact	
Gloves only	
Gown/overalls only	
Gloves & gown/overalls	
Gloves, gown/overalls & face protection (respiratory mask and goggles)	
Gloves, overalls with head protection, P2 respiratory mask and goggles	
Not sure	

Any more comments:

Scenario 11

The owner brought the animal from other area (from low land area) The primary symptoms the farmer explains are decreased appetite, diarrhoea and keratitis/ corneal opacity, intermittent fever pale mucous membranes, lacrimation, lethargy, loss of appetite, body condition and productivity and weight loss. The animals have localized swelling or inflammation and lesions on their bodies (oedema), have signs of and swollen lymph nodes. Majority of animals have progressively become emaciated with concurrent decreases in milk yield. Additionally, there have been abortions, premature births, perinatal losses.

What is the likely disease?	
What treatment (drugs) will you recommend	
How long will the treatment last	
What else will you recommend for example vitamins, deworming	
Would submit samples before treatment?	
If yes what treatment will you administer as you wait for results	
Would you notify authorities about this case?	
What will be the total cost for treating the disease?	
What will be the total cost for recommended extra products/services?	
What else would you recommend to the farmer?	

What biosecurity will you take when examining and attending the cows

None	
Hand wash after contact	
Gloves only	
Gown/overalls only	
Gloves & gown/overalls	
Gloves, gown/overalls & face protection (respiratory mask and goggles)	
Gloves, overalls with head protection, P2 respiratory mask and goggles	
Not sure	

Any more comments:

Scenario 12

The affected cows have suffered drastic weight loss, have diarrhoea, depressed appetite, and a fever. The cows have red nose with sores and crusting, mucopurulent nasal discharge; moist cough; and a rapid, shallow respiratory rate. The most affected cows have irregular breathing pattern and grunting on expiration, sensitivity to light, swollen eyelids, eye infection, depression, rapid and laboured breathing, coughing fits, extended head and neck, laterally rotated elbows, and breathing through an open mouth with projecting tongue. Affected dairy cows have significantly reduced milk production.

What is the likely disease?	
What treatment (drugs) will you recommend	
How long will the treatment last	
What else will you recommend for example vitamins, deworming	
Would submit samples before treatment?	
If yes what treatment will you administer as you wait for results	
Would you notify authorities about this case?	
What will be the total cost for treating the disease?	
What will be the total cost for recommended extra products/services?	
What else would you recommend to the farmer?	

What biosecurity will you take when examining and attending the cows

None	
Hand wash after contact	
Gloves only	
Gown/overalls only	
Gloves & gown/overalls	
Gloves, gown/overalls & face protection (respiratory mask and goggles)	
Gloves, overalls with head protection, P2 respiratory mask and goggles	
Not sure	

Any more comments:

Information sources
What is the main source of your information?

What are the main challenges with animal disease diagnosis?

What are the main challenges with animal disease treatment?
i.e , cost, quality, access,

What determines your treatment approach?
farmers ability to pay, personal relationships, the medicine available
